# Explanatory Models of Burnout Diagnosis Based on Personality Factors and Depression in Managing Nurses

**DOI:** 10.3390/jpm12030438

**Published:** 2022-03-10

**Authors:** María José Membrive-Jiménez, José L. Gómez-Urquiza, Nora Suleiman-Martos, Carolina Monsalve-Reyes, José Luis Romero-Béjar, Guillermo A. Cañadas-De la Fuente, Emilia Inmaculada De la Fuente-Solana

**Affiliations:** 1 Red Cross Nursing Center, University of Sevilla, 41009 Sevilla, Spain; mariajose.membrive@gmail.com; 2 Department of Nursing, Faculty of Health Sciences, Campus Universitario de Ceuta, University of Granada, 51001 Ceuta, Spain; jlgurquiza@ugr.es (J.L.G.-U.); norasm@ugr.es (N.S.-M.); 3 Departamento de Ciencias Sociales, Universidad Católica de La Santísima Concepción, Concepción 4030000, Chile; carolinamonsalve@ucsc.cl; 4 Statistics and Operational Research Department, University of Granada, 18071 Granada, Spain; 5 Department of Nursing, Faculty of Health Sciences, University of Granada, 18016 Granada, Spain; gacf@ugr.es; 6 Brain, Mind and Behaviour Research Center (CIMCYC), Campus Universitario de Cartuja, University of Granada, 18011 Granada, Spain; edfuente@ugr.es

**Keywords:** burnout, depression, logistic regression, managing nurses, personality risk factors

## Abstract

Nurse managers are affected by burnout due to the high degree of interaction between managers with their registered nurses. Explanatory models based on psychological, and personality related variables purvey an estimation to level changes in the three dimensions of the burnout syndrome. A categorical-response logistic ordinal regression model, supported on a quantitative, crosscutting, multicentre, descriptive study with 86 nursing managers in the Andalusian Health Service in Granada, Spain is performed for each dimension. The three models included different variables related to personality, as well as depression as the only explanatory variable included in all the models. The risk factor neuroticism was significant at population level and related to emotional exhaustion, whilst responsibility was significant in the model estimated to personal accomplishment dimension. Finally, depression was significant for the three dimensions of Burnout. This analysis provides useful information to help the diagnosis and evolution of this syndrome in this collective.

## 1. Introduction

Burnout syndrome is a prevalent problem, that has drawn the attention of academics [1,2]. Burnout arises from chronic workplace stress, which incites a negative impact in the professionals involved as well as their workplace [3].

The term ‘burnout’ was used for the first time in the 1970s, and since then, there have been many authors who have tried to describe the emotional stress which workers experienced in the workplace, in particular persons who give service to others [4,5].

Currently, the most widely accepted definition of burnout syndrome is the one described by Maslach and Jackson [6]. They defined burnout as an emotional response to chronic work stressors in people whose daily job engaged interaction with others [7]. Burnout was exemplified by three different dimensions: high emotional exhaustion (EE), high depersonalization (D), and low personal accomplishment (PA) [8,9]. This conceptualization is built on the Maslach Burnout Inventory (MBI), which is one of the most employed scales and besides that it is applied to quantify the degree of burnout experienced, as a low, medium, or high level [6].

The consequences of burnout suffering deteriorate the work environment, affecting management, and is often associated with different symptoms such as insomnia, depression or incapacity to concentrate [10].

As Maslach and Jackson [8] studied, professions which involve an elevated level of interaction between workers have more likelihood of suffering burnout. In general, health professions [11] and specifically nurse managers have composed the study subject of many investigations since these professions are at a high probability of suffering burnout [12]. This is largely due to the fact these managers are leaders who lead health professionals in their health service to improve the quality of patient care [13]. Furthermore, nurse managers, whose job consists of acting as the intermediary between staff and upper-level management [14], are often engaged in stressful and tough situations and frequently experience overload [15,16].

Many authors have investigated the multiple risk factors in nurse managers [17,18,19], such as the association between burnout and sociodemographic variables [20,21], as well as work-related variables: the type of work shift, job satisfaction, or seniority in the workplace [22]. By the same token, there has been little research aimed to delve into the position performed by psychological variables into the progress of the syndrome [23]. Certain researchers have described a susceptibility to develop burnout in managers whose personality is strong and is characterized by a highly negative affectivity as well as a marked social inhibition [24]. In contrast, it is lower when the nurse has a resistant personality and self-control of their inner environment [25]. Other authors have examined the impact of the five personality factors on the progress of this syndrome [25,26,27]. This model describes personality in terms of five basic factors: neuroticism, friendliness, responsibility, extraversion and openness to experience [28]. The neuroticism personality factor seems to be intimately linked to burnout progress, whilst the other personality factors seem to protect against burnout in managers [26,27]. Studies about burnout in healthcare professionals in Spain showed a 43.4% prevalence, with similar results, 44% in hospital nurses and, also, the influence of personality and depression factors in burnout [25,26,27,28,29]. It is important to analyze the impact that these variables have in nursing managers because they work in a different field.

Exploring which psychological variables are the most significant in predicting burnout severity [30], would be especially helpful in the organization of nursing services [18].

This research paper has the aim to discover risk factors related to personality and depression variables and measure their impact on prognosis at the different levels of each dimension of the burnout syndrome for nursing managers.

## 2. Materials and Methods

### 2.1. Study Design

A descriptive, crosscutting, multicenter study was made in Andalusia (Spain), with a sample of nurse managers.

### 2.2. Participants

The sample comprised 86 nurse managers working at the Andalusian Health Service. A convenience sampling was performed among professionals, who were classified as working at Primary Health Care Districts (25.6%) or Hospitals Services (74.4%). The mean age of the participants was 46.65 years (SD = 7.16), 58.1% of them were female.

### 2.3. Outcome Measures

The information related to socio-demographic variables such as age and sex, and the personality variables: extraversion (Ex); friendliness (Fr); responsibility (Ry); neuroticism (Nm); and openness (Op) [31], was acquired by means of an ad hoc questionnaire. The three dimensions of burnout: EE, DP, and PA [6]. The variables related to personality were measured by the NEO Five-Factor Inventory (NEO-FFI) [31], using the Spanish-language version [28].

The burnout syndrome was quantified by the Maslach Burnout Inventory (MBI), using the Spanish-language version [32]. The MBI contains 22 items, divided in three dimensions: 9 items for EE, 5 items for DP, and 8 items for PA. We applied the standards suggested in the MBI guidebook [6] to assess low, medium, or high levels of burnout, for each dimension.

### 2.4. Data Collection

After informing the hospitals, the local delegations of the Spanish nursing union (SATSE) explained to nurse managers our study purpose and managed the reception of the finalized surveys, in collaboration with the authors. The questionnaires were gathered in a non-random system from 31 public hospitals and primary health care centers, belonging to the Andalusian Health Service (SAS), in the last quarter of 2021.

Approximately a sample of 1064 people are working in the Public Health System of Andalusia as an intermediate position and dedicated to the administration and management of personnel, as well as their coordination within the Clinical Management Units. We obtained the participation of a total of 122 nurse managers in our study. One hundred questionnaires were returned, of which 86 had been fully completed (response rate: 70%).

### 2.5. Ethical Considerations

Involvement in our research was voluntary, individual and anonymous. The expected time required to end the survey was 45 min. The complete sample obtained information on the research and gave their written informed consent. This research was accepted by the Ethics Committee of the University of Granada (1961-N-21) as well as achieved in agreement with the ethical requirements of the Declaration of Helsinki [33].

### 2.6. Statistical Analysis

An ordinal logistic regression model was used [34] for every single burnout dimension, considering psychological and related to personality data as explanatory variables. These models were used to recognize which variables were really hazard factors because they involved changes among levels of burnout. A model without any interactions among the above-mentioned factors was deemed to best fit the records. This model was fitted in a stepwise way, forward-backward selection. The goodness-of-fit was compared using the probability ratio test and Stukel’s chi-squared test. The Wald’s test was used to evaluate the significance at population level of the factors that enter into the models. The strength ratios for every level regarding the adjacent level were achieved, according to the potential variations in the risk factors studied. The R Statistical Computing Software (version 4.1.1) was the computational tool used for the statistical analysis.

## 3. Results

### 3.1. Sample Description and Burnout Levels Dimensions

The descriptive study of the personality and depression variables deemed is shown in Table 1. Relevant work and seniority variables are described in Table 2.

A total 22.1% of the participants in this sample had high EE, whilst 30.2% presented a medium score for this dimension. On the other hand, 20.9% had high scores for DP and 36.0% had a medium score in this level. Finally, 14.0% presented high score for PA and 27.9% obtained a medium score. Table 3 shows the prevalence of the levels for each dimension of this syndrome.

### 3.2. Explanatory Model for Each Burnout Dimensions

Different descriptive variables associated to personality factors and depression are included in the models fitted for the different dimensions of this syndrome. In Table 4, Table 5 and Table 6 the parameters assessed for each explanatory variable included in the model are assessed, for every single dimension of the syndrome.

The Chi-square log-likelihood test for these models were X^2^(5, *N* = 86) = 89.71, *p* < 0.001 for EE; X^2^(5, *N* = 86) = 106.18, *p* ≤ 0.001 for D and X^2^(5, *N* = 86) = 96.09, *p* ≤ 0.001 for PA. Therefore, the model fit increased significantly compared to a model than solitary bears the constant in mind. The Stukel goodness-of-fit test for these models were X^2^(2, *N* = 86) = 2.49, *p* = 0.288 for EE; X^2^(2, *N* = 86) = 3.13, *p* = 0.209 for DP and X^2^(2, *N* = 86) = 2.32, *p* = 0.313 for PA. These results determined, therefore, that the models formed a good fit at population level with all dimensions.

According to the Wald test (see Table 3, Table 4 and Table 5), the variable depression (De) is significant at population level for the three models (*p* < 0.001) or dimensions. Neuroticism (Nm) is significant for the model referred to EE (*p* < 0.001) and responsibility (Ry) is significant for the model involved in level changes in the PA dimension of Burnout (*p* = 0.044). Odds ratios (see Table 3, Table 4 and Table 5) for the significant variables can be interpreted as a measure of strength related to the increasing or decreasing severity in each of the dimensions. All the considered personality related variables are discrete variables; therefore, it is not expected that there will be relevant changes with just one unit of increase of these variables (odds-ratios are close to one), but with more units of increase. In fact, if De amplified by 6 units, the odds ratio referred to turning to a great level of EE (respectively to a high level of D and PA) was 2.94 (respectively 2.83 and 2.86) times greater than if De did not increase. The same effect occurred with Nm in EE. Thus, if Nm expanded by 6 units, the odds ratio of passing to a superior level of EE was 3.16 times greater. Finally, an increase of 6 units in the Ry value produced an odds ratio of moving to a high level of 2.47 times greater for PA.

## 4. Discussion

The purpose of this research was to detect risk factors linked to personality and depression variables and measure their impact on prognosis at the different levels of each dimension of the burnout syndrome for nursing managers. Regarding the first topic and the personality related variables, three models that provide a first approximation of level changes in each dimension of burnout syndrome were obtained. These models included the variables related to the personality, neuroticism and responsibility, as well as depression, as explanatory variables. Depression (De) is a risk factor included in the three models or dimensions of Burnout. Neuroticism (Nm) is a risk factor included in the model related to EE, therefore it is involved in level changes for this dimension of the burnout syndrome. Responsibility (Ry) is involved in level changes associated to the dimensions related to PA. These models can predict the likelihood of a person at a burnout level, as appears from variations in the explanatory variables.

About the second topic, the results showed that high values of De were associated to situations of higher burnout severity in the three dimensions of burnout, with similar results in other studies [22,23], whilst Nm was associated to situations of greater burnout severity in the EE dimension of this syndrome. In the same way, Ry is a risk factor involving increasing burnout severity in PA.

Some studies inform that the appearance of De in nurse managers can be mediated by possible problems of communication with their subordinates [16] and sometimes concluding in higher level of burnout [25,35,36,37]. Furthermore, other factors such low salaries, hard workplace politics, high workloads and head chiefs demands [38], as well as lack of professional growth and insufficient resources, deteriorate the managers’ physiological condition [39], which may influence De.

Regarding the association between Nm and EE, the study carried out by De la Fuente [37], in oncology nurses, supports our findings. Nm describes as the level of emotional instability, could be an outcome of the nurse managers’ attempt to acclimatize to the stressful condition and to alleviate the pressure experienced in the work environment [25], although it can sometimes be supposed by employees as a deficiency of guidance [40] and authority [18]. Nurse managers, who suffer this type of behavior, tend to use avoidance and distraction as useless coping strategies [26]. Finally, all these strategies to cope with Nm lead to EE and if this condition is sustained, in time it could provoke the intention to leave [15].

Moreover, the association between Ry and PA could be explained by the fact that nurse managers may feel they are subjected to excessive responsibility impacting on their motivation, job satisfaction [24,41,42]. To these factors, it could be added the lack of empowerment and recognition, as well as lack of perceptions of their supervisor’s resonant leadership [15]. Then, an excessive feeling of Ry may decrease PA in nurse managers.

### 4.1. Study Limitations

This study has some limitations. The design used in this research makes it impossible to establish causal relations between results obtained. It could be solved by implementing a longitudinal design in future studies, to analyze the burnout progression in nursing managers. Other limitations are the sample selection and the group size. The sample was selected using nonrandomized methods and some groups analyzed were relatively small. Although these limitations were offset by the point that these nurses worked in numerous health centers, the subsequent results must be taken with prudence. Finally, the study did not include the influence of the pandemic as a variable.

### 4.2. Clinical Implications

Nurse managers have a fundamental role in the leadership of nursing professionals and hospital care units. Work overload, insufficient resources, having to deal with problems affecting the personnel in their charge, absence of time, and the deficiency of backing from superiors are, between others, risk factors for burnout in nursing managers [15,19]. Likewise, knowing the personality factors—such as neuroticism, depression, and responsibility—that influence the burnout development allows managers to recognize professionals at risk early, and treat this problem. Nurse managers with high risk factors should receive an early intervention base on stress-coping strategies and cognitive-behavioral therapy, to prevent [43,44] and promote the detection of burnout symptoms. This is essential in order to avoid a mishandling of employees, that could worsen the work environment for nurses and hence the quality of employment.

## 5. Conclusions

In the health sector, nurse managers are exposed to possible burnout syndrome. Nurse managers whose personality variables include high levels of neuroticism, responsibility and who suffer from depression are at higher risk of developing burnout. The professionals who present these factors should also be considered, since they are further likely to suffer emotional exhaustion, depersonalization, and emotions of low personal accomplishment.

## Figures and Tables

**Table 1:** Descriptive study of the personality and depression variables.

Variables (*N* = 86)	M	SD
Nm	26.08	6.05
Fr	46.85	4.76
Ry	48.62	5.12
Ex	44.69	6.63
Op	38.86	6.35
De	44.00	12.89

Remark: De = Depression, Nm = neuroticism, Fr = friendliness, Ry = responsibility, Ex = extraversion, Op = openness, M = mean, SD = standard deviation.

**Table 2:** Descriptive study of relevant work and seniority variables.

**Work Variables**	**Level**	**% (*N*)**
Work shift (*N* = 86)	(1) Rotating	17.4 (15)
	(2) Fixed	82.6 (71)
		
Guard shift (*N* = 86)	(1) No	51.2 (44)
	(2) Yes	48.8 (42)
		
Nurse manager before (*N* = 86)	(1) No	75.6 (65)
	(2) Yes	24.4 (21)
		
**Seniority Variables**	**Mean (SD)**	**Min–Max**
Workplace in months (*N* = 86)	84.0 (114.4)	1.0–432.0
Profession in months (*N* = 86)	276.0 (90.2)	72.0–504.0
Manager in months (*N* = 86)	54.0 (63.6)	0.0–288.0

**Table 3:** Burnout levels for every single dimension.

Levels	EE			DP			PA		
	Low	Medium	High	Low	Medium	High	Low	Medium	High
**%**	47.7	30.2	22.1	43.0	36.1	20.9	58.1	27.9	14.0
**M (SD)**	16.90 (11.17)			5.64 (5.22)			39.85 (6.89)		

Remark: EE = emotional exhaustion, DP = depersonalization, PA = personal accomplishment, M = mean, SD = standard deviation.

**Table 4:** Logit model for Emotional Exhaustion (EE) ${\hat{L}}_{s}\left(\mathrm{Nm},\mathrm{De}\right)={\hat{B}}_{0}+{\hat{B}}_{\mathrm{Nm}}\mathrm{Nm}+{\hat{B}}_{\mathrm{De}}\mathrm{De};\;s=1,2$.

*Factors*	*B*	*SD*	*Wald*	*p*	*Odds*	*CI for 95% Odds*	
						*Lower*	*Upper*
Nm	0.140	0.054	2.596	<0.001	1.150	1.137	1.163
De	0.077	0.026	2.948	<0.001	1.080	1.074	1.086

*Note*: EE = emotional exhaustion, Nm = neuroticism, De = Depression, B = parameter, SD = standard error, Wald = statistic, *p* = *p*-value, Odds = odds ratio, CI = confidence interval, Lower = lower limit of the CI, Upper = upper limit of the CI.

**Table 5:** Logit model for Depersonalization (DP) ${\hat{L}}_{s}\left(\mathrm{Fr},\mathrm{De}\right)={\hat{B}}_{0}+{\hat{B}}_{\mathrm{Fr}}\mathrm{Fr}+{\hat{B}}_{\mathrm{De}}\mathrm{De};\;s=1,2$.

*Factors*	*B*	*SD*	*Wald*	*p*	*Odds*	*CI for 95% Odds*	
						*Lower*	*Upper*
Fr	−0.084	0.048	−1.745	0.081	0.919	0.910	0.929
De	0.038	0.018	2.111	0.035	1.039	1.035	1.043

*Note*: DP = depersonalization, Fr = friendliness, De = Depression, B = parameter, SD = standard error, Wald = statistic, *p* = *p*-value, Odds = odds ratio, CI = confidence interval, Lower = lower limit of the CI, Upper = upper limit of the CI.

**Table 6:** Logit model for Personal Accomplishment (PA) ${\hat{L}}_{s}\left(\mathrm{Ry},\mathrm{De}\right)={\hat{B}}_{0}+{\hat{B}}_{\mathrm{Ry}}\mathrm{Ry}+{\hat{B}}_{\mathrm{De}}\mathrm{De};\;s=1,2$.

*Factors*	*B*	*SD*	*Wald*	*p*	*Odds*	*CI for 95% Odds*	
						*Lower*	*Upper*
Ry	−0.102	0.051	−2.011	0.044	0.903	0.893	0.913
De	0.050	0.020	2.469	0.014	1.051	1.047	1.056

*Note*: PA = personal accomplishment, Ry = responsibility, De = depression, B = parameter, SD = standard error, Wald = statistic, *p* = *p*-value, Odds = odds ratio, CI = confidence interval, Lower = lower limit of the CI, Upper = upper limit of the CI.

## Data Availability

The data presented in this study are available on request from the corresponding author.
